# Mechanisms and Physiological Roles of the CBL-CIPK Networking System in *Arabidopsis thaliana*

**DOI:** 10.3390/genes7090062

**Published:** 2016-09-08

**Authors:** Jingjing Mao, S. M. Nuruzzaman Manik, Sujuan Shi, Jiangtao Chao, Yirong Jin, Qian Wang, Haobao Liu

**Affiliations:** 1 Tobacco Research Institute, Chinese Academy of Agricultural Sciences, Qingdao 266101, China; maojingjing40@163.com (J.M.); mak2423@yahoo.com (S.M.N.M.); shisujuan2014@163.com (S.S.); chaojiangtao@caas.cn (J.C.); 2 College of Agriculture and Plant Protection, Qingdao Agricultural University, Qingdao 266109, China; 3 Dezhou Academy of Agricultural Sciences, Dezhou 253051, China; jinyirong688@163.com

**Keywords:** CBL, CIPK, signaling, physiological functions, *Arabidopsis thaliana*

## Abstract

Calcineurin B-like protein (CBL)-CBL-interacting protein kinase (CIPK) network is one of the vital regulatory mechanisms which decode calcium signals triggered by environmental stresses. Although the complicated regulation mechanisms and some novel functions of CBL-CIPK signaling network in plants need to be further elucidated, numerous advances have been made in its roles involved in the abiotic stresses. This review chiefly introduces the progresses about protein interaction, classification and expression pattern of different *CBL*s and *CIPK*s in *Arabidopsis thaliana*, summarizes the physiological roles of CBL-CIPK pathway while pointing out some new research ideas in the future, and finally presents some unique perspectives for the further study. The review might provide new insights into the functional characterization of CBL-CIPK pathway in *Arabidopsis*, and contribute to a deeper understanding of CBL-CIPK network in other plants or stresses.

## 1. Introduction

Calcium ion (Ca^2+^), acting as a ubiquitous “secondary messenger” in the cell, regulates numerous functional and developmental processes in plants [1,2]. Gene networking plays an important role in decoding diverse Ca^2+^ signals to adapt changeable environment [3]. In calcium regulatory networks, calcium sensor proteins specifically recognize Ca^2+^ signals and transmit them to downstream pathway [4]. The calcium sensor Calcineurin B-like protein (CBL), cooperating with CBL-interacting protein kinase (CIPK), occupies an essential position in stress-responsive mechanisms in plants [5,6,7]. The first CBL and CIPK member were both identified in *Arabidopsis thaliana* [8,9]. The CBL family shares sequence similarity with the B-subunit regulators of calcineurin B subunit (CNB) in yeast and neuronal calcium sensors (NCS) in animals [8]. And CIPK family is a kind of protein kinase which was considered to be functionally similar to sucrose non fermenting 1 (SNF1) in yeast and AMP-dependent kinase (AMPK) in mammalian [10].

Physiological roles of CBL and CIPK were firstly uncovered in salt overly sensitive (SOS) pathway [11]. The *Arabidopsis* mutants *sos1*, *sos2* and *sos3*, produced the same salt-sensitive phenotype under high-salt stress [12]. SOS3 and SOS2, also known as CBL4 and CIPK24 respectively, were demonstrated to synergistically up-regulate the activity of plasma membrane (PM)-located Na^+^/H^+^ exchanger SOS1 in *Arabidopsis*, leading to the Na^+^ efflux from cells in high-salt environment [11,13]. Thereafter, 10 *CBL*s and 26 *CIPK*s were identified in *Arabidopsis* [6,14].

It has been found that CBL-CIPK pathways work as regulators in nutrients transport systems, regulating sodium (Na^+^) [11,13], potassium (K^+^) [15,16], magnesium (Mg^2+^) [17], nitrate (NO_3_^−^) [18,19] and proton (H^+^) homeostasis [20]. Recently, some reviews have also drawn attention to the possible involvement of the *CBL*s and *CIPK*s in different ions sensitivity [21,22]. Focusing on the *CBL*s and *CIPK*s in *Arabidopsis*, this review elaborates the mechanisms of protein interaction, classification as well as their expression patterns, and attaches importance to introducing the physiological roles of different CBL-CIPK pathways. Some new research ideas and directions in the future are also discussed below. Unless stated, all the genes in this review are from *Arabidopsis*.

## 2. Mechanisms of CBL-CIPK Pathway

Structural characteristics of CBL and CIPK proteins provide the basis for their interaction. The crystal structure of the complex of Ca^2+^-CBL4 with the C-terminal regulatory domain of CIPK24 was firstly resolved [23]. It unveils that how the CBL-CIPK complex decodes intracellular Ca^2+^ signals triggered by extracellular stimulation [24].

The CBL protein harbors four elongation factor hands (EF-hands), and each EF hand contains a conserved α-helix-loop-α-helix structure responsible for Ca^2+^ binding [24]. The EF-hands are organized in fixed spaces which are 22, 25 and 32 amino acids distances from EF1 to EF4 in turn [25,26]. The loop region is characterized by a consensus sequence of 12 residues DKDGDGKIDFEE [14,27]. Amino acids in positions 1 (X), 3 (Y), 5 (Z), 7 (−X), 9 (−Y) and 12 (−Z) are responsible for Ca^2+^ coordination [24]. It should be noted that EF1 contains an insertion of two amino acid residues between position X and position Y [24]. Variation of amino acids in these positions causes the change of Ca^2+^-binding affinity [14]. As is known, amino acid residues of CBL4 at the positions X, Y, Z and −Z bind Ca^2+^ depending on side chain donor oxygen, while backbone carbonyl oxygen atom and water facilitation are used at positions −Y and –X, respectively [24].

**Figure 1:**
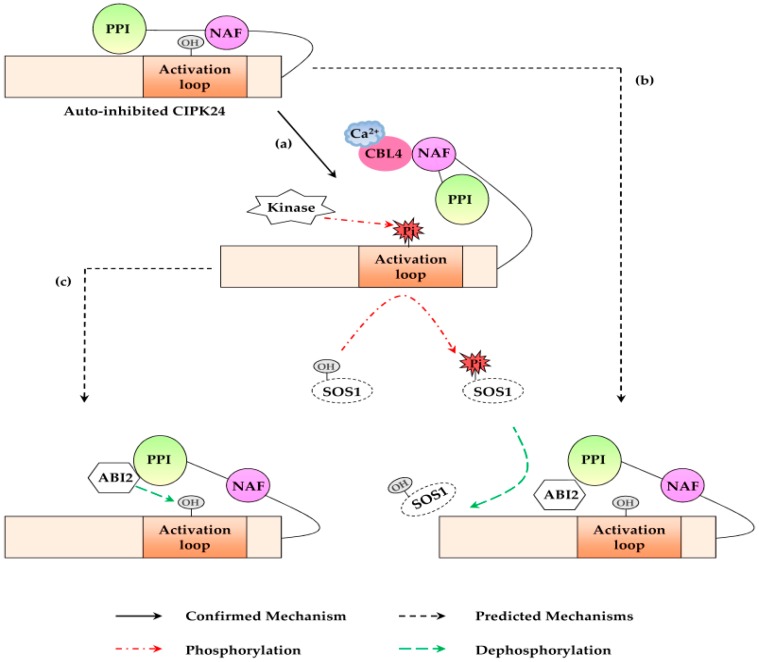
Mechanism of Calcineurin B-like protein 4 (CBL4)-CBL-interacting protein kinase (CIPK24) signaling pathway. (**a**) The Ca^2+^-binding CBL4 interacts with the NAF motif of CIPK24 and changes the conformation of CIPK24. CIPK24 exposes its activation loop and then is phosphorylated by an unknown upstream protein kinase. Activated CIPK24 phosphorylates and stimulates salt overly sensitive 1 (SOS1), subsequently; (**b**) Abscisic acid-insensitive 2 (ABI2) binds to the phosphatase interaction (PPI) domain of CIPK24 and dephosphorylates SOS1 which was phosphorylated by CIPK24; (**c**) Activated CIPK24 is dephosphorylated by ABI2 and its activity is inhibited.

The CIPK protein consists of two domains, one is the conserved N-terminal kinase catalytic domain, which encompasses a phosphorylation site-containing activation loop, the other is the highly variant C-terminal regulatory domain harboring NAF/FISL motif and a phosphatase interaction motif (PPI) [28]. The NAF motif, named by its highly conserved amino acids Asn (N), Ala (A), Phe (F), Ile (I), Ser (S) and Leu (L), is necessary for binding CBL protein [28]. This motif is necessary for sustaining the interaction between CIPK24 and CBL4, and is able to attach the C-terminal regulatory domain of CIPK24 to cover its activation loop for keeping the kinase in an auto-inhibited state (Figure 1) [28].

Attachment of Ca^2+^ by EF hands leads to the modification of molecular surface properties of CBL4 [24], and helps CBL4 interact with CIPK24 via the NAF motif. The interaction triggers the conformational changes of CIPK24 and exposes its activation loop [28]. Once the activation loop is free, the auto-inhibited CIPK24 is phosphorylated by an unknown upstream kinase and activates CIPK24. Subsequently, the activated CIPK24 phosphorylates the Na^+^/H^+^ exchanger SOS1 on the PM to exclude the excess Na^+^ from the cell (Figure 1a) [11,29]. Abscisic acid-insensitive 2 (ABI2), a member of protein phosphatase 2C (PP2C), was identified as a CIPK24-interacting phosphatase [30]. The salt-tolerant phenotype of *abi2* indicated that ABI2 is a negative regulator of CIPK24 in SOS pathway [30]. Up to now, the blocking mechanism of ABI2 in CBL4-CIPK24 pathway is not yet elucidated. It is assumed that ABI2 might function in the process of dephosphorylating SOS1 (Figure 1b) or CIPK24 (Figure 1c) [30].

## 3. Classifications and Expression Patterns of CBLs and CIPKs

**Figure 2:**
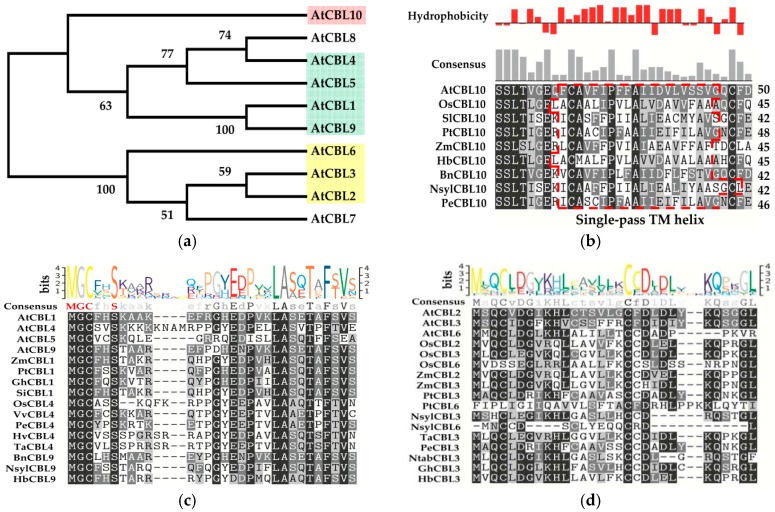
Classification and motifs display of CBLs. (**a**) Phylogenetic relationship of CBL proteins in *Arabidopsis thaliana*. The protein harboring signal-pass transmembrane (TM) helix motif, dual lipid modification MGCXXS/T motif and tonoplast targeting sequence (TTS) is marked in pink, green and yellow shades, respectively; (**b**) Detailed sequence comparisons of the single-pass TM helix of CBL10s from different plants; (**c**) Detailed sequence comparisons of the MGCXXS/T motif of CBL1, 4, 5 and 9 proteins; (**d**) Detailed comparisons of the TTS of CBL2, 3 and 6 proteins. Note: The amino acid sequences are available from UniProt (www.uniprot.org/) and we added some other CBL protein sequences from other organisms for better display. Phylogenetic tree is constructed by MEGA6 with Neighbor-Joining method. Multiple sequence alignment is provided by BoxShade (www.ch.embnet.org/software/BOX_form.html). Hydrophobicity and Consensus are indicated by Texshade. Single-pass TM helix is predicted by HMMTOP (www.enzim.hu/hmmtop/). Codification: At, *Arabidopsis thalians*; Bn, *Brassica napus*; Gh, *Gossypium hirsutum*; Hb, *Hordeum brevisubulatum*; Hv, *Hordeum vulgare*; Nsyl, *Nicotiana sylvestris*; Ntab, *Nicotiana tabacum*; Os, *Oryza sativa*; Pe, *Populous euphratica*; Pt, *Populus trichocarpa*; Ta, *Triticum aestivum*; Si, *Setaria italica*; Sl, *Solanum lycopersicum*; Vv, *Vitis vinifera*; Zm, *Zea mays*.

The phylogenetic analysis indicated that CBLs in *Arabidopsis* were divided into three groups (Figure 2a). CBL10 is the only member in the Group I, and has the longest N terminus in *Arabidopsis* CBL family [24,31]. Same with other plants CBL10s, *Arabidopsis* CBL10 also contains a conserved signal-pass transmembrane (TM) helix at its N terminus, leading to its membrane-targeting (Figure 2b) [24,31]. Confocal fluorescence microscopy analysis indicated *Arabidopsis* CBL10 localizes in PM and tonoplast, which is consistent with our expectation [31,32,33]. There are 5 members (including CBL1, 4, 5, 8 and 9) in Group II, and they have the shortest N-termini [24,31]. Most of them contain the conserved MGCXXS/T motif for dual lipid modification (Figure 2c), which helps the CBLs to anchor in the membrane [6,34]. When fused with green fluorescent protein (GFP), CBL1 and CBL9 localize in the PM while CBL4, CBL5 and CBL8 distribute diffusely throughout the plant cells [31]. Although lack of MGCXXS/T motif, the membrane targeting of CBL8 hints that an unknown mechanism might be involved in its localization [31]. Dependent on dual lipid modifications of CBL4, CBL4-CIPK6 complex effectively mediates the translocation of inward-rectifier potassium channel AKT2 from the endoplasmic reticulum (ER) membrane to the PM in a targeting pathway [35]. Based on these results above, the subcellular localization of CBLs suggests complicated intracellular interaction between CBLs and various protein factors, which might provide us with new clues to explore their biological function. Members of Group III have relatively long N-termini [24,31]. They all possess a tonoplast targeting sequence (TTS) MSQCXDGXKHXCXSXXXCF (except CBL7) [36,37]. TTS contributes to the vacuole membrane localization for CBL2, 3, and 6 proteins (Figure 2d) [31,36].

**Figure 3:**
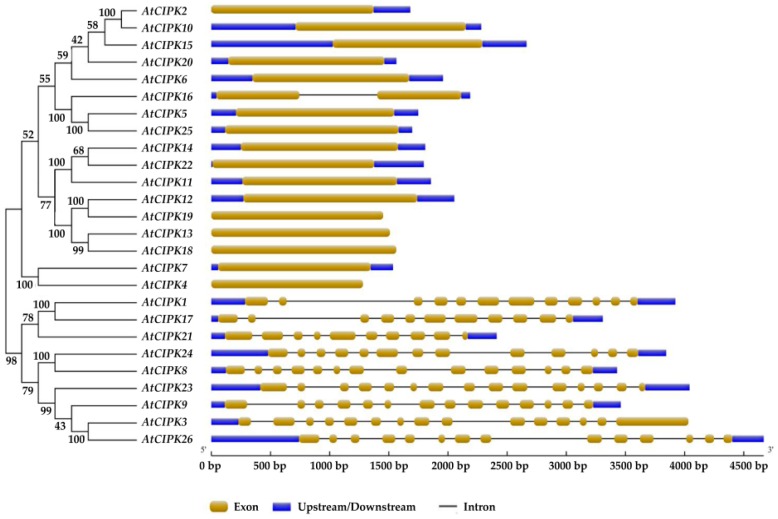
Phylogenetic analysis and intron-exon distribution of *CIPKs* in *A*. *thaliana*. The amino acid sequences are available from UniProt. The coding sequences (CDS) and genomic sequences of *CIPK*s were available from The Arabidopsis Information Resource (TAIR). It is noteworthy that only the representative splice variant was selected for the analysis as to some genes having multiple splice variants. MEGA6 with Neighbor-Joining method and online software GSDS 2.0 (gsds.cbi.pku.edu.cn/) were used for the analysis.

In our analysis, the *Arabidopsis* *CIPK* family was divided into two clades, intron-fich and intron-less clades (Figure 3). There are 17 members in the intron-less clade (including *CIPK2*, *4*, *5*, *6*, *7*, *10*, *11*, *12*, *13*, *14*, *15*, *16*, *18*, *19*, *20*, *22* and *25*), while 9 members in the intron-rich clade (inculding *CIPK1*, *3*, *8*, *9*, *17*, *21*, *23*, *24* and *26*). Segmental and tandem duplications were found to contribute to the expansion of these two clades [38]. Generally, the CIPK proteins distribute commonly in the cell, including cytoplasmic and nuclear compartments, and they can be translocated by their interacting CBLs [16,31,33,39].

**Figure 4:**
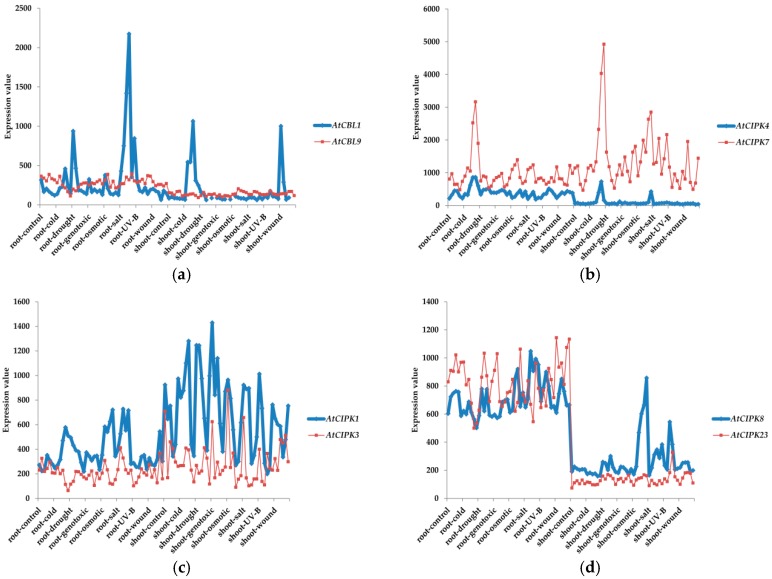

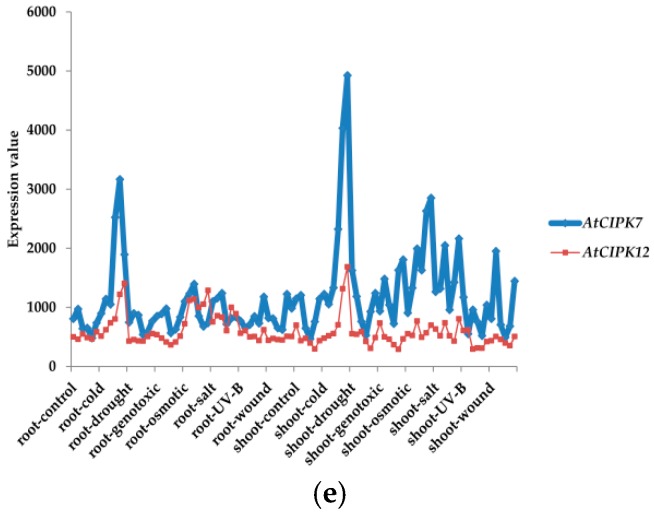
Expression pattern correlations of CBLs and CIPKs under different abiotic stress in shoots and roots. The expression data for each treatment were collected from TAIR database [41].

It is important to note that, although homologous genes are used to be considered to be functionally identical, calcium sensors with high sequence similarities may perform very distinct functions and paralogous genes usually evolve some new functions [40]. There are some paralogous genes in CBL-CIPK network, including *CBL1*/*9*, *CBL2*/*3*, *CIPK1*/*17*, *CIPK2*/*10*, *CIPK4*/*7*, *CIPK5*/*25*, *CIPK12*/*19* and *CIPK13*/*18* (Figure 2a and Figure 3). Expression data from The Arabipsis Information Resource (TAIR) database showed that there are limited correlations between the expression patterns of *CBL1* and *CBL9* (r = 0.11049, *p* = 0.2885, r and *p* represent relativity and probability, respectively), *CIPK4* and *CIPK7* (r = −0.04698, *p* = 0.6512) under some abiotic stresses (Figure 4a,b) [41]. There are also some experimental proofs indicating that *CBL1* has distinctively different functions from *CBL9* in drought and ABA responses [40,42,43]. Meanwhile, genes with a closer evolutionary relationship may have similar functions and work cooperatively to adapt to the complex and changeable environment. There are strong correlations between the expression patterns of *CIPK1* and *CIPK3* (r = 0.86272, *p* < 0.0001), *CIPK8* and *CIPK23* (r = 0.57839, *p* < 0.0001) in intron-rich clade (Figure 4c,d), *CIPK7* and *CIPK12* (r = 0.68713, *p* < 0.0001) in intron-less clade (Figure 4e).

## 4. Physiological Roles of CBL-CIPK Signaling Pathways in *Arabidopsis*

Numerous experiments were conducted to elucidate physiological roles of CBL-CIPK complexes. Taking a panoramic view of the reported progress on CBL-CIPK network, the signaling pathways can be divided into two categories, designated as plasma membrane targeting and tonoplast targeting pathways, according to the functional sites.

### 4.1. Plasma Membrane Targeting CBL-CIPK Pathways

Generally, PM targeting CBL-CIPK pathways play an indispensable role for plants to adapt to the changeable environment, by regulating the influx and efflux of ions.

#### 4.1.1. CBL1/CBL9-CIPK23 Pathways

**Figure 5:**
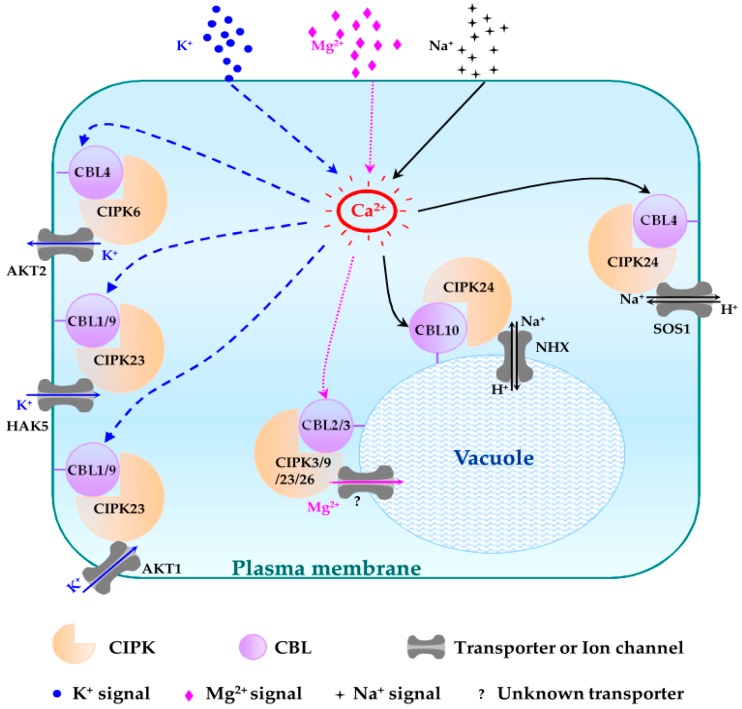
Schematic representation of different CBL-CIPK complexes and their functions in regulating sodium (Na^+^), potassium (K^+^) and magnesium (Mg^2+^) homeostasis. The regulatory pathways of K^+^, Mg^2+^ and Na^+^ are indicated in blue, pink and black lines, respectively. Question mark (?) indicates an unknown tonoplast-localized transporter.

CBL1/9-CIPK23 pathways were firstly identified to positively regulate potassium absorption [15,44]. There are two K^+^ uptake systems as the major contributors in *Arabidopsis*, the high-affinity system relying on the high-affinity K^+^ transporter 5 (HAK5) and the low-affinity system relying on *Arabidopsis* K^+^ transporter (AKT1). When the external K^+^ concentrations are below 10 μM, HAK5 is the only transporter capable of uptaking external K^+^ in *Arabidopsis* [45]. CBL1-CIPK23 complex is able to phosphorylate HAK5 and up-regulates its activity of K^+^ uptake in the roots [45]. When K^+^ concentrations are higher than 500 μM, CBL1/9-CIPK23 complexes activate inward-rectifier K^+^ channel AKT1 to absorb K^+^ by phosphorylation [15]. Between 10 and 200 μM, HAK5 mainly contributes to K^+^ uptake cooperated by AKT1 (Figure 5) [15,45].

CBL1/9-CIPK23 complexes are also involved in the abscisic acid (ABA)-related regulation of stomata aperture. The concentration of ABA is increased in drought or high-salt stressed plants. RCAR/PYR/PYL ABA receptors can sense excessive ABA and then inhibit the PP2C-type protein phosphatases ABA insensitive 1 (ABI1) and ABI2 [46]. Activity inhibition of ABI1 and ABI2 allow CBL1/9-CIPK23 complexes to phosphorylate slow anion channel associated 1 (SLAC1) and slow anion channel 1 homolog 3 (SLAH3) present in guard cells. Activated SLAC1 and SLAH3 will reduce the guard cell volume and shut down the stomata [46,47,48]. Activated anion channels increase the release of chloride and nitrate, and result in the stomatal closure finally [46,48]. These results seemed that the CBL1/9-CIPK23 complexes might play a positive role in the stomatal closure in response to ABA by increasing anion efflux of guard cell. However, Nieves-Cordones et al. [49] found that, compared to wild-type plants, *cipk23* and *akt1* adult plants lost less water and produced a more efficient stomatal closure in response to ABA and disruption of CBL1/9 or CIPK23 led to an ABA-hypersensitive and drought-tolerance phenotype [49,50]. CBL1/9-CIPK23 complexes are able to increase K^+^ influx, which tends to trigger the stomatal opening [44]. Based upon the phenotype of the mutant, it is probable that CBL1/9 and CIPK23 might have more active influence on K^+^ uptake rather than anion efflux in ABA-related regulation of stomatal movement, and there might be other kinases or transporters involved in anion efflux to sustain the ion balance. One support is the identification of open stomata 1 (OST1), a calcium-independent kinase from SNF1-related protein kinase 2 (SnRK2) family, which was found to be able to activate SLAC1 [47].

CBL1/CBL9-CIPK23 complexes regulate NO_3_^−^ uptake under the nitrate deficient condition by phosphorylating the nitrate transporter 1/peptide transporter family 6.3 (NPF6.3), which is also known as nitrate transporter 1.1 (NRT1.1) and chlorate resistant 1 (CHL1) [18,19]. NPF6.3 is a dual-affinity NO_3_^−^ transporter and it works as a high- or low-affinity transporter depending on the phosphorylation or dephosphorylation of threonine 101 (Thr101). Under nitrate-poor conditions (NO_3_^−^ < 1 mM), CBL9/CIPK23 complex switches NPF6.3 from low- to high-affinity transport activity by phosphorylating Thr101. [18]. When nitrate is rich (NO_3_^−^ > 1 mM), low-affinity transport activity of NPF6.3 is inhibited by the CBL9/CIPK23-dependent phosphorylation of Thr101, while enhanced by ABI2-dependent dephosphorylation of CBL1/9-CIPK23 complexes [19,51,52]. It is interesting to study whether phosphorylated NPF6.3 can be dephosphorylated by ABI. Moreover, Leran et al. [53] reported that NPF6.3 is not only an influx transporter participating in the uptake of NO_3_^−^ in roots, but also an outward transporter responsible for the NO_3_^−^ secretion into the xylem. And CBL9-CIPK23 complex inhibits its activity of NO_3_^−^ efflux. The molecular mechanism of the two opposite functions of NPF6.3 is not yet clarified.

#### 4.1.2. CBL1/9-CIPK26 Pathways

The study of CBL1/9-CIPK26 pathways with reactive oxygen species (ROS) illustrated a novel role of CBL-CIPK pathway in the interrelations between Ca^2+^ and ROS signaling in plants and showed the anfractuosity and flexibility of the CBL-CIPK network [54,55]. Respiratory burst oxidase homologues (RBOHs), also known as plant NADPH oxidases (NOXs), are indispensable components of the enzymatic complexes generating ROS [54]. There are 10 RBOH members (namely RBOHA to RBOHJ) in this small family, and RBOHF is one of the major members responding to biotic stresses. Ca^2+^ binding to the EF-hands of RBOHF and phosphorylation by CBL1/9-CIPK26 complexes are the pre-conditions of synergistic activation of RBOHF [55].

#### 4.1.3. CBL9-CIPK3 Pathway

CBL9-CIPK3 complex is found to be involved in the regulation process of ABA signaling [56]. However, *CIPK3* is involved in the salt-induced rather than drought-induced ABA-dependent pathway, and disruption of *CBL9* and *CIPK3* changed some marker genes’ expression in a different way when responding to ABA [40,56,57]. So it is assumed that CBL9 and CIPK3 might fulfill their function independently under some special conditions, by cooperating with other proteins. One potential interactive protein of CBL9 is CIPK15, which was reported to work with CBL1 in a common pathway as the negative regulator of ABA responses [58]. *CBL9* shares a high sequence similarity with *CBL1* [58]. Coincidently, *CBL1* didn’t respond to ABA in some researches, while the RNA interference (RNAi)-based *cbl1* used by Guo might result in the *CBL9*’s silencing [58]. Yeast two-hybrid (Y2H) experiment showed that there was no interaction between CBL9 and CIPK15 [14]. Whether CBL9 interacts with CIPK15 needs to be further detected.

#### 4.1.4. CBL2-CIPK11 Pathway

PM H^+^-ATPase (PMA), a kind of proton pump localized in the PM, is activated by phosphorylation of Threonine 947 (Thr947) and the resultant binding of 14-3-3 [59]. However, CBL2-CIPK11 complex is able to phosphorylate Serine 931 of PMA and inhibits Thr947 from binding to an upstream unknown kinase, leading to the final failure of PMA-14-3-3 interaction [20,60]. Unactivated PMA cannot develop a proton electrochemical gradient and provide a driving force for ion and metabolite transport [59]. Consistently, *cipk11* mutant plants are more tolerant to the alkaline environment [20]. In short, CBL2-CIPK11 complex plays a negative role in the activation process of PMA. 

#### 4.1.5. CBL4-CIPK6 Pathway

CBL4-CIPK6 pathway is complementary to the well-established CBL-CIPK-K^+^ channels system. Distinguishing from other CBL-CIPK-K^+^ channels pathway, CBL4-CIPK6 complex cannot phosphorylate its binding partner AKT2 in vitro, for CIPK6 C-terminal regulatory domain is enough for the interaction with CBL4 and AKT2. Especially, it is found that CBL4 is the key factor for the translocation of AKT2 complex from ER to PM, relying on its dual lipid modifications [35]. CBL4-CIPK6 complex might work as an escort and avigraph, helping the ER-retained AKT2 to move to PM to export K^+^ during both K^+^ loading in sources and K^+^ uploading in sinks [35,61]. It means that CBL4-CIPK6-AKT2 pathway is a phosphorylation-independent but Ca^2+^-dependent pathway in K^+^ regulation [35].

#### 4.1.6. CBL4-CIPK24 Pathway

As the earliest identified CBL-CIPK pathway described in the introduction, CBL4-CIPK24 pathway is mostly found to function for Na^+^ excluding in root tissues under high-salt conditions (Figure 5) [11,29]. CBL4-CIPK24 complex drives the Na^+^/H^+^ exchanger SOS1 to send Na^+^ back into soil to improve the salt tolerance of plants [29].

### 4.2. Tonoplast Targeting CBL-CIPK Pathways

Vacuolar membrane targeting CBL-CIPK pathways are mostly characterized to help cells to avoid the toxicity of harmful ions by sequestrating them into vacuole [17,32,36,62,63,64]. Interestingly, the tonoplast targeting CBL-CIPK pathway also participates in growth and development of plants [65,66].

#### 4.2.1. CBL2/3-CIPK3/9/23/26 Pathways

CBL2 and CBL3 locate in tonoplast to regulate intracellular ion homeostasis by interacting with vacuolar H^+^-ATPase (V-ATPase). *cbl2cbl3* double mutant is hypersensitive to excessive metal ions including Ca^2+^, Fe^3+^, Cu^2+^, K^+^, Zn^2+^ and Mg^2+^, except for Na^+^ [36]. Although *cbl2cbl3* displayed severe development deficiency under excessive Mg^2+^ condition, the mutant plants retained lower Mg content, indicating that CBL2 and CBL3 function in vacuolar Mg^2+^ sequestration [17]. It is probable that CBL2/3 and CIPK3/9/23/26 create a multivalent network to protect plants from high Mg^2+^ toxicity by sequestrating Mg^2+^ in vacuole (Figure 5) [17]. CBL3 and CIPK9 are also engaged in K^+^ ion distribution and translocation in plants [62,63]. Although *CBL2* and *CBL3* sharing 92% similarity in amino acid sequence and their overexpressing lines showed similar sensitive phenotype under low K^+^ condition, the two genes displayed different expression patterns and the respective mutant showed different phenotype under low K^+^ condition [36,63]. *CBL2* and *CBL3* might be a paralogous gene pair differing in some functions.

#### 4.2.2. CBL2/3-CIPK12 Pathways

Tonoplast-localized CBL2/3-CIPK12 complexes work as crucial regulators in the vacuole dynamics, pollen germination and tube growth [65]. Interestingly, *CBL2* and *CBL3* overexpressing plants displayed impaired pollen tube growth, which is similar to those of single mutant and *cbl2cbl3* double mutants. Also, overexpression of *CIPK12* induced severe vacuolar phenotypes, and loss of function of *CIPK12* led to impairment of polar growth. The results indicated that CBL2/3-CIPK12 complexes play a fundamental role during the pollen tube development, and an appropriate and balanced activity of CBL2/3-CIPK12 complexes is more crucial than their existence for pollen germination and pollen tube growth [65]. Overexpression or disruption of *CIPK19*, the closest homolog of *CIPK12*, also reduced the elongation and expansion of the pollen tube. The two genes might be functionally redundant in the growth and development of pollen tube [67].

#### 4.2.3. CBL2/3-CIPK21 Pathways

Although *cipk21* was hypersensitive to high Na^+^ and CIPK21 was detected to be co-localized with CBL2/3 in tonoplast under high Na^+^ stress, the function of CBL2/3-CIPK21 complexes in the regulation of high Na^+^ is not yet certain [64]. Moreover, *cbl2cbl3* double mutant did not present the similar salt-hypersensitive phenotype with *cipk21* [36,64]. There are two possibilities to explain such a discrepancy, one is that CBL2/3-CIPK21 complexes might do not function in the regulation of Na^+^ homeostasis, the other is that other CBL proteins located in tonoplast (such as CBL6) might play a redundancy role in response to high Na^+^ stress [64]. Further research is needed to uncover this tonoplast targeting pathway.

#### 4.2.4. CBL10-CIPK24 Pathway

As a supplement to the function of CIPK24 in response to high salt, CBL10-CIPK24 pathway set up a novel salt-tolerance mechanism. Exhibiting a similar salt hypersensitivity phenotype, *cbl10* accumulates lower Na^+^ than wild plant. CBL10 functions mainly in shoots and enhances the activity of a vacuolar Na^+^/H^+^ exchanger (NHX) for Na^+^ sequestration by interacting with CIPK24 in the case of salt toxicity (Figure 5) [32,68].

## 5. Perspectives

Considerable advances have been made in the knowledge of CBL-CIPK pathways, including the regulation in ion homeostasis, response to hormone and environmental signaling and involvement in plant growth and development. In addition, the resolution of some critical crystal structures (such as CBL2 [25], CBL4 [26], CBL4-CIPK24 complex [23] and CBL2-CIPK14 complex [69]), the identification of diverse downstream substrates (Table A1) and the revealing of correlations with other signaling pathways indeed enrich our understanding in CBL-CIPK network. However, many questions are not yet answered.

Limited crystal structures cannot cover all details in the regulatory process of CBL-CIPK network. How are the Ca^2+^ signals triggered by different environmental stress recognized? How does the same CBL-CIPK complex transmit different signals to the corresponding substrates? A typical example is that the recognition mechanism of CBL1-CIPK23 complex to its downstream substrate AKT1 under different K^+^ concentrations [15,45]. It is assumed that, the decoding of Ca^2+^ signals might rely on the EF-hands, for that the Ca^2+^-binding affinity of EF-hands with differing calcium binding ability. Moreover, CBL conformation triggered by Ca^2+^-binding might be dynamic, and result in a subsequent exposure of the activation loop of CIPKs in a different degree. Thereafter, activity of CIPK is activated fully or partly, which might also interfere with its substrates [45]. Another possibility is the case that different concentrations of Ca^2+^ caused by different degrees of environment stimulus create different kinds and expression levels of CBL-CIPK complexes as well as those of their cooperative partners. Certainly, all of these are only speculations and required to be tested by resolving more crystal structures.

The homeostasis of many ions is regulated by CBL-CIPK pathways. For example, CIPK8 regulates the homeostasis between nitrate and other anions, such as malate/fumarate or borate [70], while CIPK24 affects the homeostasis between Ca^2+^ and Na^+^ homeostasis by regulating the cation/proton exchanger 1 protein (CAX1) [71]. The studies are valuable for our understanding about plant nutrition. Plants face numerous environmental stresses during their life cycle, including biotic and abiotic stress. Although much effort is focused on unveiling the function of CBL-CIPK network in response to abiotic stresses, its roles in biotic stresses are limited. Some stress expression data showed that some members in *CBL* and *CIPK* family, including *CBL1*, *CIPK11* and *CIPK25* responded to fungus stress [72,73], and *CBL2* functions responding to bacteria [73]. Studies on the relevance of CBL-CIPK pathways and biotic stresses will make more contributions to the resistance breeding of plants. Moreover, CBLs and CIPKs also work in an independent manner. As is reported, CBL3 is able to inhibit 5′-methylthioadenosine nucleosidase 2 (MTAN2) in a CIPK-independent way [74], and CBL10 is found to interact with AKT1 without interacting with any CIPKs to regulate K^+^ homeostasis in *Arabidopsis* [75]. CBLs and CIPKs may construct a much bigger regulatory network beyond our imagination which needs to be discovered.

## Appendix

**Table A1:** Summary of reported substrates of CBL-CIPK complexes.

Substrates		CBL-CIPK Complexes Upstream	Functions of the Substrate Proteins
Ion channels	AKT1	CBL1/9-CIPK23	An inward K^+^ channel uptaking K^+^ at K^+^ concentrations higher than 10 μM [15].
	AKT2	CBL4-CIPK6	An outward K^+^ channel exporting K^+^ for the K^+^ unloading [35,61].
	SLAC1	CBL1/9-CIPK23	An anion channel involved in the ABA-regulation of stomata aperture, leading to the closure of stomata [46,47].
	SLAC3	CBL1/9-CIPK23	An anion channel involved in the ABA-regulation of stomata aperture, leading to the closure of stomata [48].
Ion transporters	CHL1	CBL1/9-CIPK23	A nitrate transporter uptaking or secreting NO_3_^−^ [18,19].
	HAK5	CBL1/9-CIPK23	A K^+^ transporter uptaking K^+^ at K^+^ concentrations below 200 μM [45].
	SOS1	CBL4-CIPK24	A Na^+^/H^+^ exchanger exporting Na^+^ back into soil [11,29].
	Vacuolar Na^+^/H^+^ antiporter	CBL10-CIPK24	A Na^+^/H^+^ antiporter sequestrating Na^+^ into vacuole [32].
Other	PM H^+^-ATPase	CBL2-CIPK11	A proton pump localized in the PM, driving ion and metabolite to transport across the PM [59,60].
	Vacuolar H^+^-ATPase	CBL2/3-CIPK3/9/23/26	A proton pump localized in the tonoplast, driving ion and metabolite to transport across the tonoplast [17,36].
	RBOHF	CBL1/9-CIPK26	A member of respiratory burst oxidase homologues which are the indispensable components of the enzymatic complexes generating ROS [54,55].
